# The Effectiveness of Pregabalin for Post-Tonsillectomy Pain Control: A Randomized Controlled Trial

**DOI:** 10.1371/journal.pone.0117161

**Published:** 2015-02-23

**Authors:** Soo Seog Park, Dong-Hyun Kim, In-Chul Nam, Il-Hwan Lee, Jae-Woong Hwang

**Affiliations:** 1 Department of Anesthesiology and Pain Medicine, College of medicine, The Catholic University of Korea, Seoul, Republic of Korea; 2 Department of Otorhinolaryngology-Head and Neck Surgery, College of medicine, The Catholic University of Korea, Seoul, Republic of Korea; The Chinese University of Hong Kong, HONG KONG

## Abstract

**Background:**

Although various analgesics have been used, postoperative pain remains one of the most troublesome aspects of tonsillectomy for patients.

**Objective:**

The aim of the present study was to evaluate the effectiveness of premedication using pregabalin compared with placebo (diazepam) on postoperative pain control in patients undergoing tonsillectomy.

**Methods:**

Forty-eight adult patients were randomly divided into a control group and a pregabalin group. Preoperatively, patients in the control group received 4 mg diazepam orally as placebo, whereas those in the pregabalin group received 300 mg pregabalin orally. All participants were provided with patient-controlled analgesia using fentanyl for 24 hours after surgery. Postoperative pain treatment included acetaminophen 650 mg three times daily for 8 postoperative days. The primary outcome measure was the total amount of patient-controlled fentanyl consumption after tonsillectomy. Secondary outcome measures were the number of injections of ketorolac tromethamine (each 30 mg) requested by patients, pain scores, overall satisfaction scores, drowsiness, nausea, dizziness, headache, and vomiting after the surgery. *P* < 0.05 was considered statistically significant.

**Results:**

The total amount of fentanyl demanded decreased significantly in the pregabalin group (*P* < 0.001). There were no significant differences in the number of ketorolac tromethamine injections, pain scores, overall satisfaction scores, drowsiness, nausea, dizziness, headache, and vomiting between the two groups.

**Conclusion:**

Administration of 300 mg pregabalin prior to tonsillectomy decreases fentanyl consumption compared with that after 4 mg diazepam, without an increased incidence of adverse effects.

**Trial Registration:**

KCT0001215

## Introduction

Tonsillectomy is one of the most common surgical procedures performed in otorhinolaryngology. However, postoperative pain following tonsillectomy is one of the most difficult to manage in this area of surgery. The severe pain from a tonsillectomy can delay the recovery process and extend the hospital stay [1]. Therefore, various analgesics and a number of surgical techniques have been suggested to improve pain relief [2–4]. Opioids or nonsteroidal anti-inflammatory drugs (NSAIDs) and local anesthesia have been used exclusively or in combination. However, opioids can cause respiratory depression, bradycardia, hypoxia, nausea, and vomiting; NSAIDs can increase the risk of bleeding in the operated area [3–5]; and local anesthesia can cause toxic reactions and upper airway closures [6]. Therefore, there is a need for a new approach that can control the pain after a tonsillectomy while reducing the use of opioids and NSAIDs.

Recent advances in the pathophysiology of pain have suggested that it is possible to prevent or attenuate the central neural hyperexcitability that contributes to enhanced postoperative pain [3,4]. Pregabalin is a novel anticonvulsant drug that has demonstrated analgesic effects in acute postoperative pain management. Jokela et al. [7] reported that perioperative administration of 600 mg pregabalin decreases oxycodone consumption compared with administration of 10 mg diazepam, but is associated with an increased incidence of adverse effects. Mathiesen et al. [8] reported that pregabalin and pregabalin combined with dexamethasone reduced postoperative pain scores and consumption of ketobemidone following tonsillectomy, although dizziness was increased with pregabalin. However, there are few studies of the effectiveness of premedication with pregabalin for post-tonsillectomy pain control compared with an active placebo, such as diazepam, to exclude the effects of sedation or dizziness. Therefore, this study was conducted to evaluate the effectiveness of pregabalin premedication compared with diazepam as an active placebo on postoperative pain control after tonsillectomy.

## Materials and Methods

### Ethics Statement

The study was approved by Institutional Review Board of Incheon St. Mary’s Hospital (IRB Number: OC10MISE0025) and the Korean Food and Drug Administration (KFDA). It was registered at clinical research information service, republic of Korea (http://cris.nih.go.kr/cris/index.jsp, registration number: KCT0001215). The purpose and method of the research were explained to the patients before the surgery, and written consent was obtained. The protocol for this trial and supporting CONSORT checklist are available as supporting information; See S1 Checklist and S1 and S2 Protocol.

### Study Design and Study Population

Patients over the age of 18 years who were scheduled for elective tonsillectomy at our hospital during the period between December 2010 and August 2012 were enrolled in this study. This was a randomized, double-blind study that was set up according to a computer-generated block randomization. Patients were excluded from the study based on the following criteria: (1) allergy to drugs in the study, (2) alcohol or drug abuse, (3) treatment with antidepressant or antiepileptic medication within 4 weeks of the operation, (4) pregnant or breastfeeding patients, (5) tonsillar malignancy, (6) impaired kidney function, or (7) daily intake of analgesics or intake of any analgesic within 24 hours of surgery.

### Study Interventions

Prior to surgery, we explained the goal of this study to all patients, who were then educated in the use of the visual analogue scale (VAS) chart and the use of a patient-controlled analgesia (PCA) pump. The night before and 1 hour before surgery each patient received either a 150 mg capsule of pregabalin (total 300 mg before surgery) (Lyrica, Freiburg, Germany) or a 2 mg capsule of diazepam orally (total 4 mg before surgery). General anesthesia was induced with intravenous propofol (2 mg/kg) and tracheal intubation was facilitated with intravenous rocuronium bromide (0.6 mg/kg). Anesthesia was maintained with sevoflurane and 50% nitrous oxide in oxygen. Paralysis was reversed with glycopyrrolate and pyridostigmine at the end of surgery. Tonsillectomy was performed using the same technique for all patients, including monopolar electrocautery; bleeding was controlled using simple compression, bipolar electrocautery, and topical application of hydrogen peroxide. As a basic analgesic regimen, both groups were given acetaminophen 650 mg three times daily (total 1950 mg per day) for 8 postoperative days. While in the hospital, patients in both groups were supplied with 1% fentanyl that was administered via a PCA device (Abbott Aim Plus^TM^, Abbott Laboratories, Illinois) with the same parameters (demand dose = 2 ml, lockout time = 10 min, no basal infusion), and the total amount of injected fentanyl was recorded before discharge. Additionally, 30 mg of ketorolac tromethamine was injected intramuscularly at the patient’s request, and the number of ketorolac tromethamine injections was recorded. The grade of pain was self-assessed by the patients using a visual analogue scale (VAS) that ranged from 0 (no pain) to 10 (most severe pain). The pain grade during resting periods (rVAS) and during swallowing (sVAS) was assessed at 1, 2, 4, 8, 12, and 24 hours postoperatively, and then daily for 7 days after discharge. Each patient was questioned regarding their overall satisfaction with the analgesic effects during the first 24 hours after surgery and again 8 days after surgery (scored from 0, not satisfied, to 10, very satisfied). Possible side effects of the medications, including drowsiness, nausea, dizziness, headache, and vomiting, were assessed and recorded in the patient’s medical chart during the 24 hours following tonsillectomy.

The primary outcome measure was patient-controlled fentanyl consumption from 0 to 24 hours after tonsillectomy. Secondary outcome measures were the number of ketorolac tromethamine injections, postoperative pain score at rest and swallowing, the overall satisfaction score, and occurrence of side effects such as drowsiness, nausea, dizziness, headache, and vomiting.

### Sample Size and Statistical Analysis

Based on data from a previous study [9], the presumption was that the total fentanyl consumption of the control group would be 47 ml (standard deviation 28). We considered a 50% reduction (24 ml) to be of clinical relevance. With a *P*-value of 0.05 and a power of 80%, a sample calculation showed that 22 patients were required in each group. To allow for dropouts, we enrolled 25 patients in each group.

Results are presented as the mean ± standard error. *P* < 0.05 was considered significant. Assumption of normality was checked using the Kolmogorov—Smirnov (K–S) test. Descriptive statistics were obtained to determine the influence of each group’s clinical parameters on the results. A χ^2^ test and a *t*-test were conducted to assess the differences in the clinical characteristics of the two groups. A Mann—Whitney *U*-test or *t*-test was used to analyze overall differences between groups. We used the *t*-test to analyze the differences in each variable between groups at each designated monitoring point of the follow-up period. Repeated measures ANOVA was used to analyze the interaction between time and group for each variable. For evaluation of side effects such as drowsiness, nausea, dizziness, headache, and vomiting, Fisher’s exact test comparing the two groups was used.

## Results

**Fig 1:**
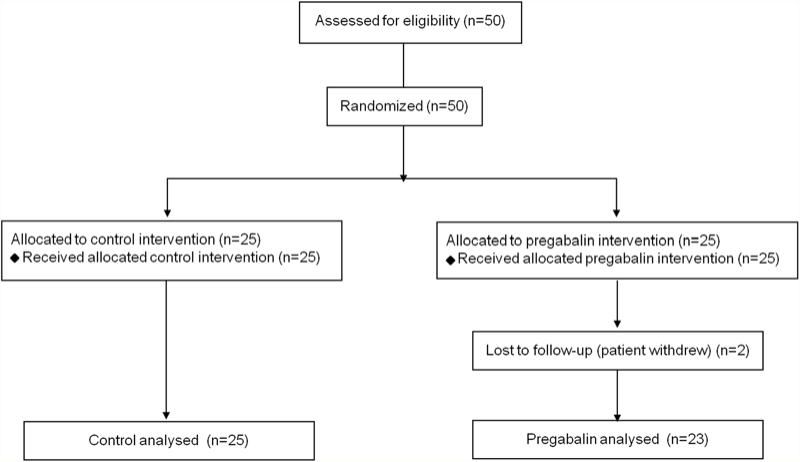
Participant flow diagram.

Fifty patients were included and were randomly assigned to a group. Two subjects were considered dropouts after initial randomization (both withdrew) and were therefore not subjected to further statistical analysis. Consequently, 48 patients were included in the final analyses (Fig. 1). As shown in Table 1, there were no significant differences between the two groups in sex, age, height, weight, time of operation, and anesthesia.

**Table 1:** Clinical characteristics of the patients.

	Control	Pregabalin	*P*
Male/Female	10/15	10/13	0.52
Age (y)	34.4 ± 2.4	32.4 ± 2.1	0.81
Height (cm)	165.1 ± 1.9	166.4 ± 2.1	0.82
Weight (kg)	64.7 ± 2.0	69.3 ± 4.3	0.89
Operation time (min)	62.4 ± 5.7	62.3 ± 6.2	0.89
Anesthesia time (min)	89.8 ± 6.7	74.4 ± 6.1	0.09

Results are presented as the mean ± standard error.

*P* value was assessed using t-test and *χ* ^*2*^ test.

**Fig 2:**
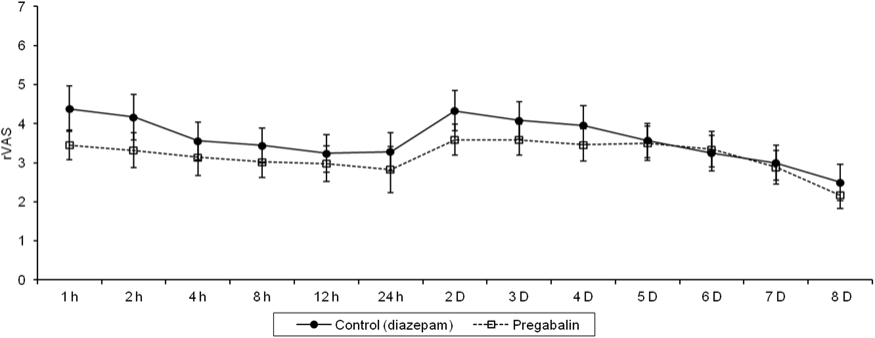
Visual analogue scale scores during rest (rVAS) after tonsillectomy in the control and pregabalin groups. Results are expressed as the mean score ± standard error (vertical bars represent standard error). There were no significant differences in the pain score between the two groups throughout the postoperative period. *Abbreviation* h: hour, D: day.

**Fig 3:**
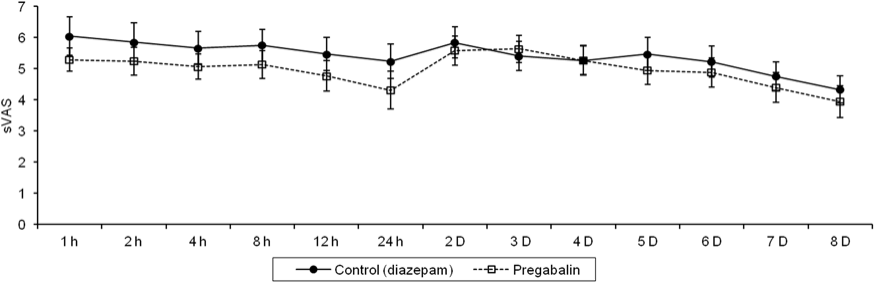
Visual analogue scale scores during swallowing (sVAS) after tonsillectomy in the control and pregabalin groups. Results are expressed as the mean score ± standard error (vertical bars represent standard error). There were no significant differences in the pain score between the two groups throughout the postoperative period. *Abbreviation* h: hour, D: day.

The total amount of PCA fentanyl used by the pregabalin group during admission was significantly lower than that used by the diazepam control group, with an 84% reduction in the pregabalin group compared with the control group (*P <* 0.001) (Table 2). The results suggest that pregabalin could reduce post-tonsillectomy pain during admission. The number of ketorolac tromethamine injections in the pregabalin group was lower than that in the control group, but the difference between the groups was not significant (Table 2). In the postoperative period, the rVAS value in the pregabalin group was lower than that in the control group at all monitoring points except on the fifth, sixth, and seventh postoperative days, but these differences were not statistically significant. There was no significant interaction between time and groups for rVAS (*P* = 0.24). We found a trend to a decrease in rVAS over time within the first 8 days postoperatively (*P* = 0.04), even though there was an increase in rVAS two days after discontinuation of PCA because of patient discharge from hospital (Fig. 2). Throughout the postoperative period, the sVAS in the pregabalin group was lower than that in the control group, except on the second, third, and fourth postoperative days; but these differences were not statistically significant. There was no significant interaction between time and groups for sVAS (*P* = 0.50). There was an increase in sVAS two days after discontinuation of PCA because of patient discharge from hospital but we could not determine whether there was a trend to decreased sVAS over time within the first 8 days postoperatively (*P* = 0.11) (Fig. 3). The overall satisfaction score for pain control was assessed at 24 hours and at 8 days after surgery in the control and pregabalin groups. There were no significant differences observed in the satisfaction score between the two groups 24 hours after tonsillectomy or 8 days after tonsillectomy. There was no significant interaction between time and groups in the overall satisfaction score (*P* = 0.34) (Table 3). Drowsiness was the most common side effect in patients from both groups, but the occurrence of this symptom was not significantly different. Dizziness was the second most common symptom, and occurred more in the control group (88%) than in the pregabalin group (78%), but this difference was not significant. Nausea and vomiting were more common in the pregabalin group, but the occurrence of these symptoms did not differ significantly between groups (Table 4).

**Table 2:** The amounts of intravenous analgesic demanded by patient-controlled analgesia (PCA) and the number of injections of ketorolac tromethamine.

	Control	Pregabalin	Difference	CI (lower)	CI (upper)	*P*
Total amount of fentanyl	62.16 ± 7.47	9.90 ± 3.45	52.25 ± 8.23	35.48	69.02	< 0.001
No. of ketorolac injections	0.87 ± 0.13	0.33 ± 0.21	0.54 ± 0.29	–0.06	1.15	0.08

Results are presented as mean ± standard error. CI: 95% confidence interval of the difference.

*P* value was assessed using the Mann—Whitney *U*-test (total amount of fentanyl) and t-test (number of ketorolac injection).

**Table 3:** Satisfaction scores at 24 hours and at 8 days after surgery in the control and pregabalin groups.

Follow-up point	Control	Pregabalin	Difference	CI (lower)	CI (upper)	*P*
24 hours	6.43 ± 0.53	6.86 ± 0.53	–0.42 ± 0.75	–1.95	1.10	0.57
8 days	6.87 ± 0.47	6.31 ± 0.41	0.52 ± 0.63	–1.95	1.10	0.41

Results are presented as mean ± standard error. CI: 95% confidence interval of the difference.

*P* value was assessed using t-test.

**Table 4:** Side effects during the first 24 hours following tonsillectomy.

		Control	Pregabalin	*P*
		Number (%)	Number (%)	
Drowsiness	No	1 (4)	0 (0)	0.54
	Moderate	17 (68)	14 (61)	
	Severe	7 (28)	9 (39)	
Nausea	No	10 (40)	6 (26)	0.56
	Moderate	12 (48)	14 (61)	
	Severe	3 (12)	3 (13)	
Dizziness	No	3 (12)	5 (22)	0.79
	Moderate	20 (80)	16 (70)	
	Severe	2 (8)	2 (8)	
Headache	No	9 (36)	10 (43)	0.88
	Moderate	15 (60)	13 (57)	
	Severe	1 (4)	0 (0)	
Vomiting	No	20 (80)	17 (74)	0.61
	Moderate	2 (8)	4 (17)	
	Severe	3 (12)	2 (9)	

*P* value was assessed using Fisher’s exact test

## Discussion

The present study was designed to evaluate the effects of pregabalin on postoperative tonsillectomy pain control and to control for the influence of sedation or dizziness. In this study, we have demonstrated that pregabalin reduced the fentanyl requirement significantly in the pregabalin group compared with the diazepam control group. Pain scores both at rest and during swallowing in the pregabalin group following tonsillectomy were lower than in the diazepam group, although a significant difference was not found. To our knowledge, this is the first study that has investigated the effectiveness of pregabalin premedication on postoperative tonsillectomy pain control compared with diazepam as an active placebo.

Pregabalin was originally developed as a spasmolytic agent and adjunct for the management of generalized or partial epileptic seizures resistant to conventional therapies [10]. The pharmacological effects of pregabalin are believed to result from its action as a ligand at the alpha-2-delta binding site associated with voltage-gated calcium channels in the central nervous system [11]. Potent binding of pregabalin at the alpha-2-delta site has been shown to reduce the depolarization-induced calcium influx at nerve terminals with a consequential reduction in the release of several excitatory neurotransmitters, including glutamate, norepinephrine, and substance P [11,12]. Pregabalin demonstrates highly predictable and linear pharmacokinetics, a profile that makes it easy to use in clinical practice. It is rapidly and extensively absorbed after oral dosing in the fasted state, with maximal plasma concentration occurring 1 hour after single or multiple doses, and steady state being achieved within 24–48 hours after repeated administration [11]. Furthermore, pregabalin has no effect on arterial pressure or heart rate [13] and exhibits an analgesic effect that is approximately 2–4 times stronger than that of gabapentin [14]. Therefore, several reports have indicated that pregabalin may be useful for the management of postoperative pain [7–9,13–15]. In the first trial investigating the postoperative analgesic effect of pregabalin, a dose of 300mg demonstrated significant pain-relieving properties for patients in dental surgery [15]. In patients undergoing spinal fusion surgery [13], two doses of 150 mg pregabalin reduced morphine consumption compared with placebo; in laparoscopic hysterectomy [7], two doses of 300 mg pregabalin reduced 24-hour postoperative oxycodone consumption; in laparoscopic cholecystectomy [9], 150 mg pregabalin reduced postoperative patient-controlled fentanyl consumption. In our study, patients received 150mg pregabalin at each of two time points before tonsillectomy. Treatment with pregabalin significantly decreased the consumption of fentanyl (*P <* 0.001), which is concordant with the findings of other studies [7–9,13] that have reported diminished opioid intake when pregabalin was used.

The postoperative side effects that accompanied the surgery when pregabalin was used as a preemptive analgesic conflict with previous results. In a consecutive report regarding laparoscopic OB/GYN surgery by Jokela et al. [7,16], the authors reported that when the dosage of pregabalin as a preemptive analgesic was less than 300 mg, it did not have postoperative side effects such as dizziness and drowsiness, but when administered at more than 600 mg, it increased the occurrence of postoperative side effects. By contrast, White et al. [17] reported that in cases of simple surgery, side effects appeared even when the pregabalin dosage was less than 300mg. In our study, oral administration of 300 mg pregabalin before surgery had a similar effect to that of 4 mg diazepam. No statistically significant difference was observed between the two groups in the occurrence of drowsiness, nausea, dizziness, headache, and vomiting (*P* > 0.05, Table 2).

The dosage and medication method of pregabalin that effectively reduce postoperative pain and minimize the adverse effects have not yet been delineated. In a study by Mathiesen et al. [8], patients received an oral dose of 300mg pregabalin 1 hour before tonsillectomy. Pregabalin reduced postoperative pain scores and consumption of ketobemidone following tonsillectomy, but dizziness was increased with pregabalin. Jokela et al. [7] reported that perioperative administration of 600 mg but not 300 mg pregabalin decreased oxycodone consumption postoperatively, as compared with 10 mg diazepam. Perioperative administration of 600 mg pregabalin was associated with an increased incidence of dizziness, blurred vision, and headache. Paech et al. [18] reported that a single preoperative dose of 100mg pregabalin was ineffective in reducing acute postoperative pain or improving recovery after minor surgery involving only the uterus. In our study, we used an oral dose of 150 mg pregabalin the night before and again 1 hour before tonsillectomy. Despite the relatively small dose of pregabalin used in this study, the total amount of fentanyl demanded was decreased in the pregabalin group, as compared with the control group given 4 mg diazepam. This result indicates that pregabalin had a significant analgesic effect on post-tonsillectomy pain, which is concordant with the study of Mathiesen et al. [8], while drowsiness, dizziness, and other side effects were similar to those from 4 mg diazepam.

To investigate purely pain control while reducing the influence of side effects such as sedation, we used diazepam as an active control. However, this study has some limitations. The measures of pain may be influenced by the patient’s compliance or subjectivity. Therefore, further studies using neurotransmitters [11,12], such as substance P, or other control regimens are necessary to clarify the precise influence of pregabalin on postoperative pain and to determine the appropriate dosage and medication method.

Although opioids play a fundamental role in the management of post-tonsillectomy pain, their use is associated with a number of side effects, including nausea, vomiting, and respiratory depression. Therefore, it is necessary to develop an effective approach to control post-tonsillectomy pain by combining treatment modalities that can block different pain mechanisms. In our study, pregabalin, which can control central neuronal pain sensitization, decreases fentanyl consumption without increasing the incidence of adverse effects compared with diazepam, which has a sedative effect. We believe that these results indicate that although pregabalin may have sedative side effects, in the context of post-tonsillectomy pain it has a more analgesic effect than a sedative effect. Therefore, we suggest that the addition of pregabalin prior to tonsillectomy may play an adjunct role in the control of post-tonsillectomy pain.

## Conclusion

The administration of 300 mg pregabalin prior to tonsillectomy decreases fentanyl consumption without increasing the incidence of adverse effects compared with 4 mg diazepam.

## Supporting Information

S1 CONSORT Checklist (DOC)

S1 Protocol Trial Protocol in Korean. (DOCX)

S2 Protocol Trial Protocol in English. (DOCX)
