# Supplementary material for: The Effectiveness of Pregabalin for Post-Tonsillectomy Pain Control: A Randomized Controlled Trial
Source: PLoS One. 2015 Feb 23;10(2):e0117161. doi: 10.1371/journal.pone.0117161 (PMC4338031; doi:10.1371/journal.pone.0117161)
Supplement: S1 Protocol — (DOCX) [file pone.0117161.s002.docx]

CONFIDENTIAL

Study No. : OC10MISE0025 Protocol Ver. 25-01

**편도 적출술 후 통증조절에 대한 Pregabalin의 효과**

Study Protocol

Study No : OC10MISE0025

**가톨릭대학교 인천성모병원 이비인후과**

Study No. : OC10MISE0025 Protocol Ver. 25-01

목차 페이지

1. 임상연구의 명칭 및 단계 -----------------------------------------------3

2. 임상연구 실시기관명 및 주소 -----------------------------------------3

3. 연구의 책임자 및 담당자/공동연구자 성명, 직위, 소속 --------------3

4. 관리약사의 성명 및 직명 -----------------------------------------------3

5. 의뢰자(기관)명 및 주소 --------------------------------------------------3

6. 임상연구의 목적(1차목적, 2차목적)--------------------------------------4

7. 임상연구의 배경 ---------------------------------------------------------4

8. 임상연구 제품(약물의 성분 명, 상품명)---------------------------------5

9. 대상질환 -----------------------------------------------------------------5

10. 피험자의 선정기준, 제외기준, 목표한 피험자의 수 및 그 근거 ----5

11. 임상연구의 기간 ------------------------------------------------------8

12. 임상연구의 방법--------------------------------------------------------8

(투여방법, 투여량, 투여기간, 병용요법에 대하여) 대조군 선정 여부 및 대조군에서의 처치방법 )

13. 관찰항목, 임상검사항목 및 관찰 검사 방법 -------------------------9

14. 중지 및 탈락 기준 ----------------------------------------------------10

15. 효과 평가기준,평가방법, 해석방법(통계분석방법) -------------------11

16. 부작용을 포함한 안전성의 평가기준 ,평가방법 및 보고방법 -------11

17. 피험자 동의서 양식 또는 면제시 면제 사유서 -----------------------11

18. 피험자보상에 대한 규약 -----------------------------------------------19

19. 임상시험후 피험자의 진료 및 치료기준 및 피험자의 안전보호에 대한 대책 --------------------------------------------------------------------------19

20. 피험자의 안전보호에 대한 대책 ---------------------------------------19

21. 증례기록서 양식---------------------------------------------------------20

22. 기타 임상연구를 안전하고 과학적으로 실시하기 위하여 필요한 사항---------------------------------------------------------------------------------26

23. 해당연구의 근거가 되는 임상문헌(참고 문헌)-------------------------26

**1.임상연구의 명칭 및 단계**

편도 적출술 후 통증조절에 대한 Pregabalin의 효과 (연구자 주도 연구)

**2.임상연구 실시기관명 및 주소**

가톨릭대학교 인천성모병원 주소: 인천시 부평구 부평 6동 665

**3. 연구의 책임자 및 담당자/공동연구자 성명, 직위, 소속**

-책임자 : 박수석 ,

●직위 : 부교수, 소속 : 가톨릭대 인천성모 마취과

-공동 연구자: 김동현,

●직위: 전임강사, 소속 : 가톨릭대 인천성모 이비인후과

**4.관리약사의 성명 및 직명**

해당 사항 없음

**5. 의뢰자(기관)명 및 주소**

해당 사항 없음

**6. 임상연구의 목적(1차목적, 2차목적)**

본 연구에서는 성인 편도 절제술 환자에서 Pregabalin의 전처치가 술 후 통증조절에 대한 효과를 알아보고자 한다.

-**일차목적은** 편도 수술후 0-24시간동안 환자가 자가 사용한 펜타닐 소비량을 비교하는 것이다.

-**이차목적은** ketorolac tromethamine 주사 횟수와 수술 후 안정시와 연하시 통증 점수과 환자 만족도, 졸림. 구역질, 어지럼증, 두통, 구토의 부작용 발현 여부이다.

**7. 임상연구의 배경**

편도 적출술은 이비인후과 영역에서 가장 흔하게 시행되는 수술 중 하나로 수술 후 따르는 통증이 심해 음식물 섭취의 저하, 탈수, 수면 장애, 수술 후 출혈의 위험성 증가 등으로 대부분의 환자에서 문제가 된다. 그래서 편도 적출술 후 통증을 감소시키기 위하여 다양한 진통제와 수술 방법의 변화가 시도되어 왔다. 현재까지 마약성 진통제, 비스테로이드성 항염제(nonsteroidal anti-inflammatory drugs) 등이 편도 적출술 후 진통제로 사용되어 왔으나 마약성 진통제는 호흡기와 중추신경계에 부작용이 있을 수 있고 비스테로이드성 항염제는 cyclooxygenase를 비선택적으로 억제하므로 혈소판 응집을 저해하여 수술 후 출혈을 증가시킬 수 있는 단점이 있다.

이러한 부작용을 줄이면서 적절한 진통 효과를 얻기 위하여 여러 약제들을 병용 사용하는 다각적 진통 방법이나, 수술절개 전에 약제를 투여하여 수술 후 통증을 감소시키는 선행 진통 방법들이 시도되고 있다.

Pregabalin은 (S)-3-(aminomethyl)-5-methylhexanoic acid 로서 항경련과 신경성 통증에 사용되는 약품이다. Agarwal A 등은 laparoscopic cholecystectomy를 시행받는 환자들에게서 pregabalin 수술 전 투여가 술 후 통증을 줄이고 술 후 fentanyl 소비를 줄인다고 보고 하였고 Freedman 등은 Augmentation mammaplasty 술 전후 pregabalin 투여가 술후 투여하지 않은 비교 군에 비교하였을 때 진통제 사용을 70% 감소 시키고 구역질을 46 % 감소시켰다고 하였다. 하지만 편도선 수술 전에 pregabalin 을 전처치만 하여 중추감작을 감소시켜 그 통증조절 효과를 확인한 연구는 아직까지 보고된 바가 없다.

**8.임상연구 제품(약물의 성분 명, 상품명)**

성분명: Pregabalin: (S)-3-(aminomethyl)-5-methylhexanoic acid

상품명 : Lyrica

**9. 대상질환**

가톨릭대학교 의과대학 인천성모병원 이비인후과에서 편도적출술을 시행 받을 만 18세 이상 환자를 대상

**10. 피험자의 선정기준, 제외기준, 목표한 피험자의 수 및 그 근거**

**-선정기준**

●가톨릭대학교 의과대학 인천성모병원 이비인후과에서 편도적출술을 시행 받을 만 18세 이상 환자를 대상으로 한다.

●본 연구에 동참하는 연구원은 본 임상시험에 관한 내용을 IRB 심의에 붙여 승인을 득한 후 연구를 개시하도록 하며, 중간에 변경사항이 발생하면 즉시 IRB에 보고한다. 본 임상시험에서는 피험자 선정 기준을 만족하고 시험에 참여하기로 자발적으로 동의한 피험자를 대상으로 한다.

**-제외기준**

1.약물 알러지 기왕력,

2.알코올 중독이나 약물 남용자,

3.항우울제나 항경련제 사용중인 자,

4.임신이나 모유 수유중인 자,

5.편도암,

6.신장 기능이상자,

7.매일 진통제를 복용하거나 수술전 진통제 복용하는 환자

**-목표로 하는 피험자의 수 및 근거**

기존의 참고 문헌을 토대로 하여 대조군의 총 fentanyl 소비량은 47 ml(표준편차 28)일 것으로 예상되며 약물의 효과가 있음은 약 50% 이상 총 fentanyl소비를 줄였을 때로 판정한다.

-유의 수준 (level of significance). α = 0.05

-검정력 (power)는 80 % , β = 0.2

-(μ_c_ – μ _t_) : 24

-표준 편차 δ: 28

2(Z_α/2_ +Z_β_)^2^ δ ^2^

N = --------------- = 21.366

(μ_c_ – μ _t_)^2^

※ **최소 각군당 22명은** 넘어야 한다.

(참고 문헌 : Agarwal A, Gautam S, Gupta D, Agarwal S, Singh PK, Singh U (2008) Evaluation of a single preoperative dose of pregabalin for attenuation of postoperative pain after laparoscopic cholecystectomy. Br J Anaesth 101:700-704.)

그러므로 연구 중 불가항력적으로 제외될 수도 있는 사람 (drop)을 예비로 두어 대조군 40명, pregabalin 실험군 40명으로한다.

**11. 임상연구의 기간**

-2010년 5월 1일 - 2010년 11월 30일:본원 및 식약청 IRB 통과

-2010년 12월 1일 - 2012년 8월 30일 : data 수집

-2012년 9월 1일 - : 통계 분석 및 논문 작성

**12.임상연구의 방법**

**(투여방법, 투여량, 투여기간, 병용요법에 대하여) 대조군 선정 여부 및 대조군에서의 처치방법)**

-연구 방법

●환자들은 컴퓨터를 이용해 만든 난수표를 이용하여 무작위 방법으로 실험군과 대조군으로 구분한다.

(1)실험군 : 수술 전 저녁 8시 및 수술 바로 1 시간 전 2번에 걸쳐 Pregabalin 150mg을 경구로 분할 투여한다. (총 300mg 투여)

(2)대조군 : 수술 전 저녁 8시 및 수술 바로 1 시간 전 2번에 걸쳐 diazepam 2mg (총 4mg)을 경구로 분할 투여한다.

●수술 후 통증 조절을 위해 pregabalin군과 대조군 공통으로 IV-PCA (Abbott Aim Plus^TM^)를 장치하고 Fentanyl(구연산 펜타닐, 하나제약) 1mg을 증류수 100cc와 혼합하여 정맥로로 연결한다. 환자가 단추를 누를 때 마다 2 ml가 주입되게 하며, 한번 단추를 누른 후 10분간은 작용되지 않도록 한다. 퇴원 전 투여 된 IV-PCA Fentanyl의 총 사용량을 측정하여 기록한다. 환자가 추가 진통을 원할 경우에는 ketorolac tromethamine 30mg 을 근주하고 그 횟수를 기록한다

**-마취 및 수술 방법**

●수술은 기관 삽관을 통한 전신 마취 하에서 시행하고 마취는 Fentanyl 등 마약성 제제를 제외한 것으로 동일하게 시행한다.

●수술은 전기 소작기를 이용하여 수술하고 수술 시 출혈은 단순한 압박 처치, 전기 소작법, H2O2 도포법을 사용하여 지혈한다.

**13. 관찰항목, 임상검사항목 및 관찰 검사 방법**

**- 환자 정보 기록지**

●환자의 연구병원 number, 성별, 나이, 키, 체중, 수술을 시행한 날짜, 수술명, 수술 및 마취 시간을 기록한다.

●퇴원 전 투여 된 IV-PCA Fentanyl의 총 사용량을 측정하여 기록한다. 환자가 추가 진통을 원할 경우에는 ketorolac tromethamine 30mg을 근주하고 그 횟수를 기록한다.

**-환자 설문지**

●통증 지표는 10 cm visual analog scale(VAS)을 이용하여 환자가 느끼는 통증을 0점(통증 없음)에서 10점(극심한 통증)까지 기록하고 수술 후 하루까지는 1, 2, 4, 8, 12, 24시간에, 퇴원 후에는 7 일동안 1일 1회 안정시와 연하시의 통증 수준을 기록하도록 교육한다.

●환자가 느끼는 전반적인 통증 조절에 대한 만족도를 술 후 1병일과 7병일째 VAS (0점 : 매우 불만족, 10점: 매우 만족)를 이용하여 조사하여 비교한다.

●입원 기간 동안 및 퇴원 후 1주일까지 오심, 구토, 어지러움, 졸림, 두통이 있었던 경우 이를 기록하여 약물 부작용의 발현율을 점검 한다.

**14. 중지 및 탈락 기준**

●환자는 연구중단에 대한 불이익 또는 이익의 손실 없이 언제든지 연구를 중단할 수 있다.

●또한 연구자는 환자에게 가장 최선이라고 판단하거나,

●환자가 본 임상연구의 준수 사항을 어긴 경우 환자의 동의없이 언제라도 환자를 임상연구에서 탈락시킬 수 있다. (예를 들어 설문지를 제대로 작성이 안되는 경우 등 )

●더불어 본 연구의 의뢰자가 전체 연구를 중단시킬 수도 있다.

●만일 환자가 본 연구에 참여함으로써 상해가 질병을 얻는 경우, 의학적 치료가 제공될 것이다. 상황에 따라 치료비용은 무상으로 처리될 수도 있다.

**15. 효과 평가기준,평가방법, 해석방법(통계분석방법)**

●Fentanyl 총 사용량이 적을수록, 추가 진통 ketorolac tromethamine 30mg 근 주 횟수가 적을수록 효과가 있을 것이다.

● 환자가 느끼는 전반적인 통증 조절에 대한 만족도 VAS 가 높을수록, 통증 지표가 낮을수록 효과가 있을 것으로 판단됨.

●통계처리는 SPSS를 사용할 것이며 student’s t-test나 Mann–Whitney *U*-test 시행하고 유의수준은 0.05로 할 것임

**16. 부작용을 포함한 안전성의 평가기준 ,평가방법 및 보고방법**

Pregabalin 술전 투여로 인한 부작용이나 후유증이나 합병증은 기존에 보고된 논문들에 의하면 졸음 등 외에는 거의 없을 것으로 사료되며 대개 일시적으로 보고 되었으나 본 시험기간 중 임상 시험 책임자는 환자의 안전에 만전을 기할 것이며 중대한 이상 반응 발생 시는 임상시험 심사위원회(IRB)에 보고 할 것임

**17. 피험자 동의서 양식**

**피험자 동의서**

## 1.임상연구제목: 편도 적출술 후 통증조절에 대한 Pregabalin의 효과

## 2.연구책임자: 박수석, 공동연구자 : 김동현

저희 연구진은 당신이 건강한 성인 (만18세 이상) 본원에서 편도 적출술 예정이기에 본 임상연구에 참여하시기를 권합니다.

## 3.연구 목적의 임상시험에 관하여 알아야 할 사항

- 연구진 중 한 명이 연구목적의 임상시험에 대하여 설명하여 드릴 것입니다.
- 연구목적의 임상시험은 당신이 자발적으로 참여할 수 있습니다.
- 참여 여부는 전적으로 당신의 선택에 달려 있습니다.
- 당신은 본 연구목적의 임상시험에 참여하지 않기로 결정할 수 있습니다.
- 당신은 지금 본 연구목적의 임상시험에 참여하기로 결정할 수도 있고, 추후에 결정할 수도 있습니다.
- 어떠한 결정을 하든 당신에게 불이익은 없습니다.
- 참여에 관한 결정을 내리기 전에 의문 사항에 대하여 충분히 문의하십시오.

## 4.누구에게 문의할 수 있나요?

문의, 염려, 불만 사항이 있거나 혹은 본 연구로 인해 상해를 입지 않을까 걱정되는 경우에는 저희 연구진과 상의하여 주십시오.

연락처 : 032-510-5602

본 연구는 가톨릭 대학교 인천성모병원 임상시험심사위원회의 심사 및 승인을 받았습니다. 당신은 본 연구에 관해 아래의 사항을 가톨릭중앙의료원 연구윤리사무국을 통해 가톨릭 대학교 인천성모병원 임상시험심사위원회와도 상의할 수 있습니다.

- 연구진에 의해 답변이 이루어지지 않은 문의, 염려, 불만 사항이 있는 경우
- 연구진과 접촉이 용이하지 않은 경우
- 연구진 외의 누군가와 이야기하고 싶은 경우
- 임상연구 피험자로서의 권리에 관해 문의사항이 있는 경우
- 본 임상연구에 대해 정보를 얻고자 하거나, 의견을 제시하고자 하는 경우
- 가톨릭중앙의료원 연구윤리사무국 : (02) 2258 - 7864
- 가톨릭 대학교 인천성모병원 임상시험심사위원회 : 032)280-5371

## 5.본 연구의 목적은 무엇입니까?

편도 적출술은 이비인후과 영역에서 가장 흔하게 시행되는 수술 중 하나로 수술 후 따르는 통증이 심해 음식물 섭취의 저하, 탈수, 수면 장애, 수술 후 출혈의 위험성 증가 등으로 대부분의 환자에서 문제가 됩니다. 그래서 편도 적출술 후 통증을 감소시키기 위하여 다양한 진통제와 수술 방법의 변화가 시도되어 왔습니다. 현재까지 마약성 진통제, 비스테로이드성 항염제(nonsteroidal anti-inflammatory drugs) 등이 편도 적출술 후 진통제로 사용되어 왔으나 마약성 진통제는 호흡기와 중추신경계에 부작용이 있을 수 있고 비스테로이드성 항염제는 억제하므로 혈소판 응집을 저해하여 수술 후 출혈을 증가시킬 수 있는 단점이 있습니다. 이러한 부작용을 줄이면서 적절한 진통 효과를 얻기 위하여 여러 약제들을 병용 사용하는 다각적 진통 방법이나, 수술절개 전에 약제를 투여하여 수술 후 통증을 감소시키는 선행 진통 방법들이 시도되고 있습니다. 최근 여러 수술에서 pregabalin 이 술 후 통증조절에 효과가 있다는 보고들이 발표되고 있습니다. 저희 병원에서도 편도 수술에 pregabalin 선처치가 도움이 될 수 있는지 알아보고자 합니다.

## 6.총 연구기간은 얼마나 됩니까?

연구에 참여하시는 경우, 예상되는 참여기간은 수술 전후 해서 약 1주간 입니다.

## 7.연구의 절차는 어떻게 되나요?

Pregabalin군은 수술 전 저녁 8시 및 수술 바로 1 시간 전 2번에 걸쳐 2번에 걸쳐 Pregabalin이란 약물을 복용하게 됩니다. 수술 전 저녁 8시 및 수술 바로 1 시간 전 2번에 걸쳐 Diazepam 이란 약을 복용합니다. 수술은 기관 삽관을 통한 전신 마취 하에서 시행하게 되고 마취는 마약성 제제를 사용하지 않을 것이며 모든 환자에서 동일한 방법으로 시행될 것입니다.편도 절제시 전기 소작기를 이용하여 수술하고 수술 시 출혈은 단순한 압박 처치, 전기 소작법, H_2_O_2_ 도포법을 사용하여 지혈할 것입니다. 모든 환자는 수술 후 1일째 퇴원하고 퇴원 1주일 후 외래에서 추적 관찰할 예정입니다. 수술 후 통증 조절을 위해 Pregabalin군과 대조군 공통으로 PCA(Abbott Aim Plus^TM,^, Abbott laboratories, Illinois)를 장치하여 통증을 조절해 드릴 것이며 퇴원 전에 투여 된 PCA 의 총 사용량을 측정할 것입니다. 환자가 추가 진통을 원할 경우에는 ketorolac tromethamine 30 mg을 근주하고 투여된 총 횟수를 기록할 것입니다. 또한 기본적인 진통제로 양군에서 acetoaminophen 를 동일하게 술 후 7일 동안 투여할 것입니다.

환자 분에게 드리는 설문지를 통해 통증 수치로 10 cm 표를 이용하여 환자 분이 느끼시는 통증을 0점(통증 없음)에서 10점(극심한 통증)까지 기록하실 것이고 수술 후 1, 2, 4, 8, 12, 24 시간에, 퇴원 후 7 일 동안은 1일 1회 안정시와 음식을 삼키실 때의 통증 정도를 기록하실 것입니다. 또한 환자 분이 느끼시는 전반적인 통증 조절에 대한 만족도를 술 후 1병일과 8병일째 10 cm 표를 이용하여 (0점 : 매우 불만족, 10점: 매우 만족)를 이용하여 기록하실 것입니다. 입원 기간 동안 및 퇴원 후 1주일까지 오심, 구토, 어지러움, 졸음, 두통이 있었던 경우 이를 말씀해 주시기 바랍니다. 만일 이러한 시험에 참여하시지 않으시면 기존 방식대로 수술전 아무 약도 먹지 않을 것이며 수술은 기관 삽관을 통한 전신 마취 하에서 시행하게 되고 편도 절제시 전기 소작기를 이용하여 수술하고 수술 시 출혈은 단순한 압박 처치, 전기 소작법, H_2_O_2_ 도포법을 사용하여 지혈할 것입니다. 술 후 환자가 추가 진통을 원할 경우에는 ketorolac tromethamine 30 mg을 근주하고 또한 기본적인 진통제로 양군에서 acetoaminophen 를 동일하게 술 후 7일 동안 투여할 것입니다.만일 이에 대한 문의사항들이 있으시면 032)510-5602 으로 전화하시면 성심껏 상담하여 주겠습니다.

## 8.임상연구에 참여하면서 내가 지켜야할 사항이 있습니까?

환자분이 추가 진통을 원할 경우에는 ketorolac tromethamine 30 mg을 근주하고 투여된 총 횟수를 기록할 것이니 진통제가 원하시면 말씀을 꼭 해주시길 바랍니다.환자 분에게 드리는 설문지를 통해 통증 수치로 10 cm 표를 이용하여 환자 분이 느끼시는 통증을 0점(통증 없음)에서 10점(극심한 통증)까지 기록하실 것이고 수술 후 1, 2, 4, 8, 12, 24 시간에, 퇴원 후 7 일 동안은 1일 1회 안정시와 음식을 삼키실 때의 통증 정도를 기록하실 것입니다. 또한 환자 분이 느끼시는 전반적인 통증 조절에 대한 만족도를 술 후 1병일과 8병일째 10 cm 표를 이용하여 (0점 : 매우 불만족, 10점: 매우 만족)를 이용하여 기록하실 것입니다. 입원 기간 동안 및 퇴원 후 1주일까지 오심, 구토, 어지러움, 졸음, 두통이 있었던 경우 이를 말씀해 주시기 바라며 설문지에 대한 기록을 충실히 하여 주시길 바랍니다.

**9.임상연구에 참여함으로써 예상되는 위험성이나 불편사항들은 무엇입니까?**

- 신체적 위험성 (예, 약물부작용) : 드묾

경우에 따라 구토, 구역질, 어지러움, 졸림, 두통등이 있을 수 있으나 대개 일시적입니다.

- 정신적 위험성 (예, 임상연구에 대한 거부감, 두려움 등) : 극히 드묾
- 개인정보의 위험성 (예, 개인정보의 공개 위험) : 극히 드묾
- 법적 위험성 (예, 이전 범죄력으로 고발될 가능성) : 극히 드묾
- 사회적 위험성 (예, 사회적 차별 가능성) : 극히 드묾
- 경제적 위험성 (예, 임상연구 참여로 인한 비용 부담 가능성, 보험 해약 위험성, 실직의 위험성 등) : 극히 드묾

## 10.연구참여를 원하지 않는 경우 다른 대체방법

본 연구에 참여하지 않는 경우, 귀하께서 선택하실 수 있는 다른 대체 방법은 다음과 같습니다.

만일 이러한 시험에 참여하시지 않으시면 기존 방식대로 수술전 아무 약도 먹지 않을 것이며 수술은 기관 삽관을 통한 전신 마취 하에서 시행하게 되고 편도 절제시 전기 소작기를 이용하여 수술하고 수술 시 출혈은 단순한 압박 처치, 전기 소작법, H_2_O_2_ 도포법을 사용하여 지혈할 것입니다. 술 후 환자가 추가 진통을 원할 경우에는 ketorolac tromethamine 30 mg을 근주하고 또한 기본적인 진통제로 양군에서 acetoaminophen 를 동일하게 술 후 7일 동안 투여할 것입니다.

## 11.개인정보 보호 관련 사항

연구 참여기록과 의무기록 등을 포함한 귀하의 개인정보는 이러한 정보 검토를 담당하는 관련자에게만 제공되도록 관리될 것입니다만, 완벽하게 비밀이 유지된다는 보장은 하기 어렵습니다. 임상시험심사위원회를 포함한 관련 기관, 식약청, 보건 복지부는 귀하의 자료를 열람하여 확인하고, 복사할 수도 있습니다. 연구결과는 차후 출판될 수 있으나 귀하의 이름과 다른 신상에 대한 정보는 기밀로 유지될 것입니다.

## 12.임상시험의 참여가 중지되는 경우

귀하는 연구중단에 대한 불이익 또는 이익의 손실 없이 언제든지 연구를 중단할 수 있습니다. 만일 도중에라도 연구참여를 중단하고 싶다면 연구자에게 연락하시기 바랍니다. 해당 연구자는 연구참여를 중단하는 절차를 진행할 것입니다.

또한 연구자는 귀하에게 가장 최선이라고 판단하거나, 귀하가 본 임상연구의 준수 사항을 어긴 경우 귀하의 동의없이 언제라도 귀하를 임상연구에서 탈락시킬 수 있습니다. (예를 들어 설문지를 제대로 작성이 안되는 경우 등 ) 더불어 본 연구의 의뢰자가 전체 연구를 중단시킬 수도 있음을 알려드립니다.

귀하의 건강, 복지에 영향을 주거나 지속적인 연구 참여에 영향을 주는 새로운 정보 발생시 귀하에게 알려드리겠습니다.

만일 귀하가 본 연구에 참여함으로써 상해가 질병을 얻는 경우, 의학적 치료가 제공될 것입니다. 상황에 따라 치료비용은 무상으로 처리될 수도 있습니다. 더 많은 정보가 필요한 경우 연구자에게 연락하시기 바랍니다.

**동의서**

| **연구제목: 편도 적출술 후 통증조절에 대한 Pregabalin의 효과**  아래 당신의 서명은 이 연구에 참여함을 허락하며 당신의 보호된 건강 정보가 노출되고 사용될 것임을 의미합니다. | | |
| --- | --- | --- |
|  |  | |
| 피험자 이름 |  |  |
|  |  |  |
| 피험자 서명 |  | 날짜 |
| **이 날짜 이후로는 서명하지 마십시오.** | 🡪 |  |
|  |  |  |
| 동의를 얻는 사람의 서명 |  | 날짜 |
|  |  | |
| 동의를 얻는 사람의 이름 |  |  |

**18. 피험자보상에 대한 규약**

본 연구에 참여하는 것에 대해 참가자들에 대한 금전적 보상은 원칙적으로 없다.

**19. 임상시험후 피험자의 진료 및 치료기준 및 피험자의 안전보호에 대한 대책**

피험자는 편도 수술 후 약 2주간 외래를 통한 경과 관찰 및 진료를 시행할 것임. 또한 퇴원 후 기본적인 진통제로 양군에서 동일하게 술 후 매일 투여할 예정이며 Pregabalin 술 전 투여로 인한 부작용이나 후유증이나 합병증은 기존에 보고된 논문들에 의하면 졸음 등 외에는 거의 없을 것으로 사료되며 대개 일시적으로 보고 되었으나 본 시험기간 중 임상 시험 책임자는 환자의 안전에 만전을 기할 것이며 중대한 이상 반응 발생 시는 임상시험 심사위원회(IRB)에 보고 할 것임.

**20. 기타 임상연구를 안전하고 과학적으로 실시하기 위하여 필요한 사항**

해당 사항 없음

**21. 증례기록서 양식**

수술 후 통증 기록지(환자용)

No

안녕하십니까?

편도수술을 받느라 걱정이 많으시죠?

편도수술은 수술부위가 노출되어 있어 말할 때나 식사할 때마다 통증이 생기게 되는 수술입니다. 저희 병원 이비인후과에서는 편도수술 후 환자들의 통증을 조금이라도 더 줄여드리고자 여러분들의 편도수술 후 통증에 대하여 조사하고 있습니다. 효과적인 통증조절을 위하여 시행하는 설문이니 힘드시더라도 성의껏 답변을 작성해주시면 감사하겠습니다. **입원 중 통증이 심할 때는 언제라도 담당 주치의나 담당 간호사님께 추가 진통제를 달라고 말씀해 주십시오.**

1. 통증의 정도는

0점은 전혀 아프지 않을 때, 1-2점은 불편한 정도일 때,

3-4점은 조금 아플 때. 5-6점은 꽤 많이 아플 때.

7-8점은 몹시 심하게 아플 때, 9-10점은 상상할 수 있는 가장 극심한 통증(팔, 다리가 떨어져 나가는 듯한)이 있을 때를 말합니다.

# 수술 1 시간 후 ( : )

| 통증정도 | 0 | 1 | 2 | 3 | 4 | 5 | 6 | 7 | 8 | 9 | 10 |
| --- | --- | --- | --- | --- | --- | --- | --- | --- | --- | --- | --- |
| 가만히 있을 때 |  | | | | | | | | | | |
| 침을 삼킬 때 |  | | | | | | | | | | |

# 수술 2 시간 후 ( : )

| 통증정도 | 0 | 1 | 2 | 3 | 4 | 5 | 6 | 7 | 8 | 9 | 10 |
| --- | --- | --- | --- | --- | --- | --- | --- | --- | --- | --- | --- |
| 가만히 있을 때 |  | | | | | | | | | | |
| 침을 삼킬 때 |  | | | | | | | | | | |

# 수술 4 시간 후 ( : )

| 통증정도 | 0 | 1 | 2 | 3 | 4 | 5 | 6 | 7 | 8 | 9 | 10 |
| --- | --- | --- | --- | --- | --- | --- | --- | --- | --- | --- | --- |
| 가만히 있을 때 |  | | | | | | | | | | |
| 침을 삼킬 때 |  | | | | | | | | | | |

# 수술 8 시간 후 ( : )

| 통증정도 | 0 | 1 | 2 | 3 | 4 | 5 | 6 | 7 | 8 | 9 | 10 |
| --- | --- | --- | --- | --- | --- | --- | --- | --- | --- | --- | --- |
| 가만히 있을 때 |  | | | | | | | | | | |
| 침을 삼킬 때 |  | | | | | | | | | | |

# 수술 12 시간 후 ( : )

| 통증정도 | 0 | 1 | 2 | 3 | 4 | 5 | 6 | 7 | 8 | 9 | 10 |
| --- | --- | --- | --- | --- | --- | --- | --- | --- | --- | --- | --- |
| 가만히 있을 때 |  | | | | | | | | | | |
| 침을 삼킬 때 |  | | | | | | | | | | |

# 수술 24 시간 후 ( : )

| 통증정도 | 0 | 1 | 2 | 3 | 4 | 5 | 6 | 7 | 8 | 9 | 10 |
| --- | --- | --- | --- | --- | --- | --- | --- | --- | --- | --- | --- |
| 가만히 있을 때 |  | | | | | | | | | | |
| 침을 삼킬 때 |  | | | | | | | | | | |

**2. 수술한 후 퇴원하실 때까지 전반적인 통증 조절에 대하여 얼마나 만족하십니까?**

( 0점은 전혀 만족하지 못한 경우, 10점은 완전히 만족한 경우입니다.)

| 만족 정도 | 0 | 1 | 2 | 3 | 4 | 5 | 6 | 7 | 8 | 9 | 10 |
| --- | --- | --- | --- | --- | --- | --- | --- | --- | --- | --- | --- |
| 가만히 있을 때 |  | | | | | | | | | | |

**3. 다른 증상이 있는지 말씀하여 주세요.**

# 속이 미식거리고 토할 것 같았다.---- 전혀 아니다( )

좀 그렇다( )

매우 그렇다( )

# 구토를 했다.------------------- 전혀 아니다( )

좀 그렇다( )

매우 그렇다( )

# 어지러웠다.-------------------- 전혀 아니다( )

좀 그렇다( )

매우 그렇다( )

# 나도 모르게 졸리고 자고 싶었다.---- 전혀 아니다( )

좀 그렇다( )

매우 그렇다( )

# 두통이 있었다.------------------ 전혀 아니다( )

좀 그렇다( )

매우 그렇다( )

퇴원 후 통증 기록지(환자용)

Unit No :

1. 통증의 정도는

0점은 전혀 아프지 않을 때, 1-2점은 불편한 정도일 때,

3-4점은 조금 아플 때. 5-6점은 꽤 많이 아플 때.

7-8점은 몹시 심하게 아플 때, 9-10점은 상상할 수 있는 가장 극심한 통증(팔, 다리가 떨어져 나가는 듯한)이 있을 때를 말합니다.

# 퇴원 1일째 아침 식사 전 ( 일)

| 통증정도 | 0 | 1 | 2 | 3 | 4 | 5 | 6 | 7 | 8 | 9 | 10 |
| --- | --- | --- | --- | --- | --- | --- | --- | --- | --- | --- | --- |
| 가만히 있을 때 |  | | | | | | | | | | |
| 침을 삼킬 때 |  | | | | | | | | | | |

# 퇴원 2일째 아침 식사 전 ( 일)

| 통증정도 | 0 | 1 | 2 | 3 | 4 | 5 | 6 | 7 | 8 | 9 | 10 |
| --- | --- | --- | --- | --- | --- | --- | --- | --- | --- | --- | --- |
| 가만히 있을 때 |  | | | | | | | | | | |
| 침을 삼킬 때 |  | | | | | | | | | | |

# 퇴원 3일째 아침 식사 전 ( 일)

| 통증정도 | 0 | 1 | 2 | 3 | 4 | 5 | 6 | 7 | 8 | 9 | 10 |
| --- | --- | --- | --- | --- | --- | --- | --- | --- | --- | --- | --- |
| 가만히 있을 때 |  | | | | | | | | | | |
| 침을 삼킬 때 |  | | | | | | | | | | |

# 퇴원 4일째 아침 식사 전 ( 일)

| 통증정도 | 0 | 1 | 2 | 3 | 4 | 5 | 6 | 7 | 8 | 9 | 10 |
| --- | --- | --- | --- | --- | --- | --- | --- | --- | --- | --- | --- |
| 가만히 있을 때 |  | | | | | | | | | | |
| 침을 삼킬 때 |  | | | | | | | | | | |

# 퇴원 5일째 아침 식사 전 ( 일)

| 통증정도 | 0 | 1 | 2 | 3 | 4 | 5 | 6 | 7 | 8 | 9 | 10 |
| --- | --- | --- | --- | --- | --- | --- | --- | --- | --- | --- | --- |
| 가만히 있을 때 |  | | | | | | | | | | |
| 침을 삼킬 때 |  | | | | | | | | | | |

# 퇴원 6일째 아침 식사 전 ( 일)

| 통증정도 | 0 | 1 | 2 | 3 | 4 | 5 | 6 | 7 | 8 | 9 | 10 |
| --- | --- | --- | --- | --- | --- | --- | --- | --- | --- | --- | --- |
| 가만히 있을 때 |  | | | | | | | | | | |
| 침을 삼킬 때 |  | | | | | | | | | | |

# 퇴원 7일째 아침 식사 전 ( 일)

| 통증정도 | 0 | 1 | 2 | 3 | 4 | 5 | 6 | 7 | 8 | 9 | 10 |
| --- | --- | --- | --- | --- | --- | --- | --- | --- | --- | --- | --- |
| 가만히 있을 때 |  | | | | | | | | | | |
| 침을 삼킬 때 |  | | | | | | | | | | |

**2. 수술한 후 지금까지 전반적인 통증 조절에 대하여 얼마나 만족하십니까?**

( 0점은 전혀 만족하지 못한 경우, 10점은 완전히 만족한 경우입니다.)

| 만족 정도 | 0 | 1 | 2 | 3 | 4 | 5 | 6 | 7 | 8 | 9 | 10 |
| --- | --- | --- | --- | --- | --- | --- | --- | --- | --- | --- | --- |
| 가만히 있을 때 |  | | | | | | | | | | |

**성인 편도 적출술 정보 기록지(의료진용)**

No :

성별 나이 :

키 : 몸무게 :

수술일자 : 수술명 :

수술시간 : 마취시간 :

# 추가 진통제사용 여부와 횟수 :

# PCA 사용량 ( ) ml

22.기타 임상연구를 안전하고 과학적으로 실시하기 위하여 필요한 사항

해당사항 없음

**23. 해당연구의 근거가 되는 임상문헌(참고 문헌)**

1.[Agarwal A](http://www.ncbi.nlm.nih.gov/pubmed?term=%22Agarwal%20A%22%5BAuthor%5D&itool=EntrezSystem2.PEntrez.Pubmed.Pubmed_ResultsPanel.Pubmed_RVAbstract), [Gautam S](http://www.ncbi.nlm.nih.gov/pubmed?term=%22Gautam%20S%22%5BAuthor%5D&itool=EntrezSystem2.PEntrez.Pubmed.Pubmed_ResultsPanel.Pubmed_RVAbstract), [Gupta D](http://www.ncbi.nlm.nih.gov/pubmed?term=%22Gupta%20D%22%5BAuthor%5D&itool=EntrezSystem2.PEntrez.Pubmed.Pubmed_ResultsPanel.Pubmed_RVAbstract), [Agarwal S](http://www.ncbi.nlm.nih.gov/pubmed?term=%22Agarwal%20S%22%5BAuthor%5D&itool=EntrezSystem2.PEntrez.Pubmed.Pubmed_ResultsPanel.Pubmed_RVAbstract), [Singh PK](http://www.ncbi.nlm.nih.gov/pubmed?term=%22Singh%20PK%22%5BAuthor%5D&itool=EntrezSystem2.PEntrez.Pubmed.Pubmed_ResultsPanel.Pubmed_RVAbstract), [Singh U](http://www.ncbi.nlm.nih.gov/pubmed?term=%22Singh%20U%22%5BAuthor%5D&itool=EntrezSystem2.PEntrez.Pubmed.Pubmed_ResultsPanel.Pubmed_RVAbstract). Evaluation of a single preoperative dose of pregabalin for attenuation of postoperative pain after laparoscopic cholecystectomy. [Br J Anaesth.](javascript:AL_get(this,%20'jour',%20'Br%20J%20Anaesth.');) 2008 Nov; 101(5):700-4.

2.Mathiesen O, Jacobsen LS, Holm HE, Randall S, Adamiec-Malmstroem L, Graungaard BK, Holst PE, Hilsted KL, Dahl JB. [Pregabalin and dexamethasone for postoperative pain control: a randomized controlled study in hip arthroplasty.](http://www.ncbi.nlm.nih.gov/pubmed/18653493?itool=EntrezSystem2.PEntrez.Pubmed.Pubmed_ResultsPanel.Pubmed_RVDocSum&ordinalpos=2) Br J Anaesth. 2008 Oct;101(4):535-41.

3.Mathiesen O, Rasmussen ML, Dierking G, Lech K, Hilsted KL, Fomsgaard JS, Lose G, Dahl JB. Pregabalin and dexamethasone in combination with paracetamol for postoperative pain control after abdominal hysterectomy. A randomized clinical trial. Acta Anaesthesiol Scand. 2009 Feb;53(2):227-35.

4.Freedman BM, O'Hara E. [Pregabalin has opioid-sparing effects following augmentation mammaplasty.](http://www.ncbi.nlm.nih.gov/pubmed/19083556?itool=EntrezSystem2.PEntrez.Pubmed.Pubmed_ResultsPanel.Pubmed_RVDocSum&ordinalpos=1) Aesthet Surg J. 2008 Jul-Aug;28(4):421-4.

5.Jokela R, Ahonen J, Tallgren M, Haanpää M, Korttila K. [Premedication with pregabalin 75 or 150 mg with ibuprofen to control pain after day-case gynaecological laparoscopic surgery.](http://www.ncbi.nlm.nih.gov/pubmed/18448418?itool=EntrezSystem2.PEntrez.Pubmed.Pubmed_ResultsPanel.Pubmed_RVDocSum&ordinalpos=2) Br J Anaesth. 2008 Jun;100(6):834-40.

6.Ha SL, Kang BH, Lee SH, Kim SY. Postoperative results in adult patients after tonsillectomy. Korean J Otolaryngol-Head Neck Surg 1999;42:1279-83.

7.Moiniche S, Romsing J, Dahl JB, Tramer MR. Nonsteroidal antiinflammatory drugs and the risk of operative site bleeding after tonsillectomy: a quantitative systematic review. Anesth Analg 2003;96:68–77.

8.Marret E, Flahault A, Samama CM, Bonnet F. Effects of postoperative nonsteroidal antiinflammatory drugs on bleeding risk after tonsillectomy. Anesthesiology 2003;98:1497–502.

9. Grass JA, Sakima NT, Valley M, Fisher K, Jackson C, Walsh P, et al. Assessment of ketorolac as an adjuvant to fentanyl patient-controlled epidural analgesia after radical retropubic prostatectomy. Anesthesiology 1993;78:642-8.

10. Cho KS, Choi CJ, Im SK, Cha HE. Scuralfate and Guaiazulene in alleviating post-tonsillectomy morbidity. Korean J Otolaryngol-Head Neck Surg 1994;37:998-1003.

11.Carr DB, Goudas LC. Acute pain. Lancet 1999; 353: 2051–58.

12. Coderre TJ, Katz J, Vaccarino AL, Melzack R. Contribution of central neuroplasticity to pathological pain: review of clinical and experimental evidence. Pain 1993;52:259-85.

13. Goa KL, Sorkin EM. Gabapentin: A review of its pharmacological properties and clinical potential in epilepsy. Drug 1993;46:409-47.

14. Woolf CJ, Chong MS. Preemptive analgesia-treating postoperative pain by preventing the establishment of central sensitization. Anesth Analg 1993; 77: 362–79.
