# Supplementary material for: The Effectiveness of Pregabalin for Post-Tonsillectomy Pain Control: A Randomized Controlled Trial
Source: PLoS One. 2015 Feb 23;10(2):e0117161. doi: 10.1371/journal.pone.0117161 (PMC4338031; doi:10.1371/journal.pone.0117161)
Supplement: S2 Protocol — (DOCX) [file pone.0117161.s003.docx]

CONFIDENTIAL

Study No. : OC10MISE0025 Protocol Ver. 25-01

**The Effectiveness of Pregabalin for Post-tonsillectomy Pain Control**

Study Protocol

Study No : OC10MISE0025

Department of Otorhinolaryngology-Head and Neck Surgery, Incheon St. Mary’s Hospital, College of medicine, The Catholic University of Korea

Study No. : OC10MISE0025 Protocol Ver. 25-01

Content Page

1. Title of clinical study and phase-----------------------------------------------------------3

2. Clinical study institution and address-----------------------------------------------------3

3. Names, position, and department of principal investigator and subinvestigator/

Coinvestigator-----------------------------------------------------------------------------------3

4. Name and position of managing pharmacist---------------------------------------------3

5. Name of sponsor and address--------------------------------------------------------------3

6. Purpose of clinical study (primary purpose and secondary purpose)-----------------4

7. Background of clinical study---------------------------------------------------------------4

8. Clinical study product (name of ingredient, product name of the drug)--------------5

9. Indication-------------------------------------------------------------------------------------5

10. Inclusion and exclusion criteria for subjects, targeted number of subjects, and their rationale-----------------------------------------------------------------------------------6

11. Duration of clinical study-----------------------------------------------------------------7

12. Method of clinical study------------------------------------------------------------------8

13. Observation items, clinical laboratory test items, and observation methods------9

14. Discontinuation and termination criteria----------------------------------------------10

15. Efficacy evaluation criteria, evaluation method, and interpretation method (statistical analysis method)-----------------------------------------------------------------11

16. Safety evaluation criteria, evaluation method, and reporting method including side effects--------------------------------------------------------------------------------------11

17. Informed consent form or exemption statement if exempted-----------------------11

18. Regulations on subject compensation------------------------------------------------18

19. Subjects’ treatment, treatment criteria, and safety protection measures after the clinical study----------------------------------------------------------------------------------18

20. Actions for safety and protection of subjects----------------------------------------18

21. Case report form-------------------------------------------------------------------------19

22. Other necessities for safe and scientific conduct of the clinical study-----------25

23. Clinical literature that can be used as a source for the current study (references)-----------------------------------------------------------------------------------------------------25

**1.Title of clinical study and phase**

The effectiveness of pregabalin for post-tonsillectomy pain control (Investigator-Initiative Study)

**2. Clinical study institution and address**

Incheon St. Mary’s Hospital, College of Medicine, the Catholic University of Korea

665 Bupyeong 6 dong, Bupyeong-gu, Incheon, Korea

**3. Names, position, and department of principal investigator and subinvestigator/coinvestigator**

-Principal investigator: Soo Seog Park,

●Position: Associate Professor, Department: Incheon St. Mary’s Hospital, College of Medicine, the Catholic University of Korea

-Coinvestigator: Dong Hyun Kim,

●Position: Instructor, Department: Incheon St. Mary’s Hospital, College of Medicine, the Catholic University of Korea

**4. Name and position of managing pharmacist**

Not applicable

**5. Name of sponsor and address**

Not applicable

**6. Purpose of clinical study (primary purpose and secondary purpose)**

The aim of the present study was to evaluate the effectiveness of premedication using pregabalin compared with placebo (diazepam) on postoperative pain control in patients undergoing tonsillectomy.

-**The primary outcome measure** was patient-controlled fentanyl consumption from 0 to 24 hours after tonsillectomy.

-**Secondary outcome measures** were the number of ketorolac tromethamine injections, postoperative pain score at rest and swallowing, the overall satisfaction score, and occurrence of side effects such as drowsiness, nausea, dizziness, headache, and vomiting.

**7. Background of clinical study**

Tonsillectomy is one of the most frequently performed surgeries in otolaryngology. It causes problems through reduced food intake, dehydration, sleep disturbance, and increased risk of postoperative bleeding in most patients because of intense pain after the operation. Therefore, to reduce post-tonsillectomy pain, various analgesics and changes in operating methods have been attempted. Until now, narcotic analgesics and nonsteroidal anti-inflammatory drugs have been used as post-tonsillectomy analgesics. However, narcotic analgesics may have undesirable respiratory and central nervous system side effects, and nonsteroidal anti-inflammatory drugs may inhibit cyclooxygenase nonselectively to reduce platelet aggregation, increasing postoperative hemorrhage.

To reduce such side effects and obtain adequate analgesia, a multimodal analgesic regimen, which concomitantly uses multiple drugs or preemptive analgesic methods in which drugs are administered before surgery to reduce postoperative pain, will be investigated. Pregabalin, (*S*)-3-(aminomethyl)-5-methylhexanoic acid, is used for anticonvulsant and neurogenic pain. Agarwal et al. reported that preoperative administration of pregabalin reduced pain and postoperative consumption of fentanyl in patients undergoing laparoscopic cholecystectomy, and Freedman et al. stated that the administration of pregabalin before and after augmentation mammaplasty reduced the use of analgesics by 70% and vomiting by 46% compared with a control group in which these drugs were not administered before the surgery. However, there has been no study of pregabalin pretreatment before tonsillectomy in an effort to reduce central sensitization and identify the effect of its pain control.

**8. Clinical study product (name of ingredient, product name of the drug)**

Pregabalin: (*S*)-3-(aminomethyl)-5-methylhexanoic acid

Product name: Lyrica

**9. Indication**

Patients older than 18 years who will undergo tonsillectomy in the Department of Otolaryngology, Incheon St. Mary’s Hospital, College of Medicine, the Catholic University of Korea.

**10. Inclusion and exclusion criteria for subjects, targeted number of subjects, and their rationale**

**-Inclusion criteria**

●Patients older than 18 years who will undergo tonsillectomy in the Department of Otolaryngology, Incheon St. Mary’s Hospital, College of Medicine, the Catholic University of Korea will be the subject of the study.

●The investigator who manages this study will obtain approval from the IRB before starting the study. If changes occur during the study, he or she will report them to the IRB immediately. Those patients who satisfy the inclusion criteria and voluntarily consent to participate in this study are the participants

**-exclusion criteria**

1. Drug-allergic anamnesis.

2. Patients who are alcohol or drug abusers.

3. Patients who are currently using antidepressants or anticonvulsants.

4. Pregnant or lactating female patients.

5. Patients with tonsillar cancer.

6. Patients with renal dysfunction.

7. Patients who take analgesics every day or who take analgesics before the operation

**-Targeted number of participants and rationale:**

Based on the existing references, the total fentanyl consumption of the control group is expected to be 47 mL (SD 28 mL), and effectiveness of the drug is judged to be a decrease in total fentanyl consumption of more than 50%.

- Level of significance: α = 0.05

- Test power is 80%, β = 0.2

- (μ_c_ – μ_t_): 24

- Standard deviation δ: 28

2(Z_α/2_ +Z_β_)^2^ δ^2^

N = --------------- = 21.366

(μ_c_ – μ_t_)^2^

**※ There should be at least 22 subjects in each group**.

(Reference: Agarwal A, Gautam S, Gupta D, Agarwal S, Singh PK, Singh U (2008) Evaluation of a single preoperative dose of pregabalin for attenuation of postoperative pain after laparoscopic cholecystectomy. Br J Anaesth 101:700–704.)

Therefore, by allowing for those who may drop out of the study for unforeseen reasons, there should be 40 subjects in the control group and 40 subjects in the pregabalin group.

**11. Duration of clinical study**

-May 1, 2010–November 30, 2010: Passed the IRBs of our hospital and KFDA

-December 1, 2010–August 30, 2012: Data collection

-September 1, 2012– : Statistical analysis and manuscript preparation

**12. Method of clinical study**

**(administration method, administration dose, administration duration, and combination therapy), control group selection and treatment method in control group**

**-Study method**

●Patients will be divided into the study and control groups using a table of random numbers.

(1) Study group: at 8 p.m. on the day before the surgery and 1 hour before the surgery, pregabalin 150 mg will be orally administered (a total of 300 mg).

(2) Control group: at 8 p.m. on the day before the surgery and 1 hour before the surgery, diazepam 2 mg will be orally administered (a total of 4 mg).

●After tonsillectomy surgery, to control pain, IV patient-controlled analgesia (PCA; Abbott Aim Plus) will be used for both the pregabalin group and control group patients. Fentanyl (fentanyl citrate, Hana Pharm. Co.) 1 mg will be mixed with 100 cc of distilled water and connected to the intravenous access. On patient demand by button press, 2 ml will be administered, but then will not be available for 10 minutes after each administration. The total use of IV-PCA fentanyl administered before discharge will be measured and recorded. If a patient wants additional analgesic, intramuscular injections of ketorolac tromethamine 30 mg will be provided. The number of times it is administered will be recorded.

**-Anesthesia and surgical method**

●The surgery will be conducted under general anesthesia after intubation, and analgesia will be similarly maintained excluding narcotic medicines such as fentanyl, which are administered intravenously (IV).

●The surgery will be effected by electrocautery, and bleeding during the operation will be arrested by simple pressure, electrocautery, and H_2_O_2_ painting.

Pain control in patients

**13. Observation items, clinical laboratory test items, and observation methods**

**- Patient information record**

●The hospital number for each participant, their sex, age, height, body weight, date of surgery, name of surgery, operation and anesthesia time will be recorded.

●The total use of IV-PCA fentanyl that is administered before discharge will be measured and recorded. If the patient wants additional analgesic, intramuscular injections of ketorolac tromethamine 30 mg will be provided. The number of times it is administered will be recorded.

**- Patient questionnaire**

●On the pain index, which is a scale from 0 (no pain) to 10 (extreme pain), the pain the patient feels will be recorded using a 10 cm visual analog scale (VAS).

●The patient will be trained so that the resting and swallowing pain levels will be recorded at 1, 2, 4, 8, 12, and 24 hours after the operation and once a day for 7 days after discharge.

●The satisfaction regarding overall pain control that the patients feel will be investigated and compared using a VAS (0: very unsatisfactory, 10: very satisfactory) at days 1 and 8 after the operation.

●During hospitalization and up to a week after discharge, if patients feel nausea, vomiting, dizziness, drowsiness, or headache, the symptoms will be recorded and any occurrence of drug side effects will be examined.

**14. Discontinuation and termination criteria**

●Patient may discontinue patient’s participation in the study whenever patient wants without any disadvantage or loss of benefit.

●The investigator may drop patient from the clinical study at any time without patient consent if he or she judges that discontinuation is the best choice for patient.

●If patient you violate the participant compliance criteria (e.g. if patient does not complete the questionnaire).

●In addition, the sponsor of this clinical study may discontinue the entire study.

●If patient acquires any injury or disease as a result of participating in this study, we will provide appropriate medical treatment. Depending on the situation, this treatment may be free of charge.

**15. Efficacy evaluation criteria, evaluation method, and interpretation method (statistical analysis method)**

●If the total use of fentanyl is low, and the number of times the additional analgesic ketorolac tromethamine 30 mg is administered by intramuscular injection is small, the pretreatment will be considered effective.

●If the satisfaction score on the VAS for overall pain that patients feel is high and the pain index is low, the pretreatment will be considered effective.

●Statistical analysis will be conducted using SPSS, and Student’s *t* test or a Mann–Whitney *U* test will also be conducted. The level of significance will be set at 0.05.

**16. Safety evaluation criteria, evaluation method, and reporting method including side effects**

Except for drowsiness, side effects, after effects, or complications because of preoperative administration of pregabalin should not occur, according to previous reports. Nevertheless, the principal investigator will ensure the safety of subjects, and if serious adverse events occur, he or she will report them to the IRB.

**17. Informed consent form or exemption statement if exempted**

**Consent form**

## 1.Title: The effectiveness of pregabalin for post-tonsillectomy pain control

## 2.Principal investigator: Soo Seog Park, coinvestigator: Dong Hyun Kim

Our research staff recommend that you to participate in this clinical study because you are a healthy adult (18 years of age or more) and planning to undergo tonsillectomy in our hospital.

## 3.Things to know about the purpose of the clinical study

- One of our research staff will explain the purpose of this clinical study.
- Participation in this clinical study is voluntary.
- The decision to participate should be made solely by you.
- You may decide not to participate in this clinical study.
- You may decide to participate in this clinical study now or later.
- You will not be disadvantaged, whatever you decide.
- Before deciding to participate, please ask any questions you may have.

## 4.Whom should I ask?

If you have any questions, doubts, complaints, or concerns about injury from this study, please discuss them with our research staff.

Phone number:( 032)510-5602

This study has been reviewed and approved by the Catholic University of Korea Incheon St. Mary’s Hospital Institutional Review Board (IRB). You may consult the Catholic University of Korea Incheon St. Mary’s Hospital IRB through the Catholic Medical Center IRB:

- When you have any questions, concerns, or complaints that have not been answered by research staff.
- When you have difficulty making contact with research staff.
- When you would like to talk with someone other than research staff.
- When you have questions about the rights of a subject in the clinical study.
- When you attempt to receive information regarding this clinical study or provide your opinion on it.

Contact details are as follows.

- Catholic Medical Center (CMC) IRB: (02) 2258-7864
- Catholic University of Korea Incheon St. Mary’s Hospital IRB: (032) 280-5371

## 5.What is the purpose of this study?

Tonsillectomy, one of the most frequently performed operations in the field of otolaryngology, causes a decline in food intake, dehydration, sleep disturbance, and increased risk of postoperative bleeding in most patients because of intense pain after the operation. Therefore, to reduce post-tonsillectomy pain, various analgesics and changes in operating methods have been attempted. Up until now, narcotic analgesics and nonsteroidal anti-inflammatory drugs have been used for post-tonsillectomy analgesics. However, narcotic analgesics may have respiratory and central nervous system side effects, and nonsteroidal anti-inflammatory drugs may inhibit cyclooxygenase nonselectively to reduce platelet aggregation, increasing postoperative hemorrhage. To reduce such side effects and obtain adequate analgesia, a multimodal analgesic regimen that concomitantly uses multiple drugs or preemptive analgesic methods in which drugs are administered before surgery to reduce postoperative pain has been attempted. In recent operations, some studies suggest that pregabalin is effective for postoperative pain control. Our hospital is attempting to examine whether pretreatment with pregabalin can be of help in tonsillectomy.

## 6.How long is the total study period?

If you participate in this study, the expected participation period will be about a week, including preoperative and postoperative periods.

## 7.What is the procedure for this study?

The subjects in the pregabalin group will take a drug called pregabalin at 8 p.m. on the day before the operation and 1 hour before the operation. The subjects in the control group will take a drug called diazepam at 8 p.m. on the day before the operation and 1 hour before the operation. The operation will be conducted under general anesthesia through intubation, narcotic agents will not be used for anesthesia, and all patients will undergo the same method of operation. When performing the tonsillectomy, electrocautery will be used. The bleeding during the operation will be arrested by using simple pressure treatment, electrocautery, and H_2_O_2_ painting. All patients are expected to be discharged at day 1 after the operation and receive follow-up at the outpatient clinic a week after their discharge. To control postoperative pain, patients in both the pregabalin and control groups will be equipped with a patient-controlled anesthesia device (PCA; Aim Plus, Abbott Laboratories, Illinois), and the total amount of fentanyl administered will be measured before discharge. If a patient wants additional analgesics, intramuscular injections of ketorolac tromethamine 30 mg will be provided, and the total number of times of injection will be recorded. As a basic analgesic, acetaminophen will be administered identically to patients in both groups for 7 days after the operation.

In the questionnaire that we will give you, you will record the degree of pain you feel from 0 (no pain) to 10 (extreme pain) using a 10 cm table at 1, 2, 4, 8, 12, and 24 hours after the operation, and once a day for 7 days after discharge while resting and after swallowing. You will also record the satisfaction you feel regarding overall pain control by using a 10 cm table (0, very unsatisfactory and 10, very satisfactory) at days 1 and 8 after the operation. If you experience nausea, vomiting, dizziness, drowsiness, or headache during the hospitalization or up to 7 days after discharge, please tell us. If you do not participate in this study, you will not be given any drugs before the operation as is currently normal, and the operation will be performed under general anesthesia in the same way, with the same postoperative care. If you have any questions regarding this operation, please call us on (032)510-5602***.***

## 8.What should I observe while participating in the clinical study?

Please( tell us if you want additional analgesics. If so, we will provide intramuscular injections of ketorolac tromethamine 30 mg. In the questionnaire that we will give you, you will record the degree of pain you feel from 0 (no pain) to 10 (extreme pain) using a 10 cm table at 1, 2, 4, 8, 12, and 24 hours after the operation, and once a day for 7 days after discharge while resting and after swallowing. You will also record the satisfaction you feel regarding overall pain control by using a 10 cm table (0, very unsatisfactory and 10, very satisfactory) at days 1 and 8 after the operation. If you experience nausea, vomiting, dizziness, drowsiness, or headache during the hospitalization or up to 7 days after discharge, please tell us. Please respond to the questionnaire truthfully.

## 9. What are some expected risks or discomforts if I participate in the clinical study?

- Physical risk (e.g. adverse drug effect): rare

Vomiting, nausea, dizziness, drowsiness, or headache may occur, but these discomforts are usually only temporary.

- Mental risk (e.g. resistance to clinical study, fear, etc.): extremely rare
- Risk of personal information (e.g. risk of private information exposure): extremely rare
- Legal risk (e.g. possibility of being prosecuted in relation to previous criminal history): extremely rare
- Social risk (e.g. possibility of social discrimination): extremely rare
- Economic risk (e.g. burden of expenses because of participation in the clinical study, risk of insurance contract cancellation, risk of unemployment, etc.): extremely rare

## 10.Alternative options if you do not want to participate in the clinical study

If you do not want to participate in this clinical study, alternative options you may choose are as follows.

If you do not participate in this study, you will not be given any drugs before the operation, as is currently normal. The operation will be performed under general anesthesia through intubation, electrocautery will be used for the tonsillectomy, and hemorrhage during the operation will be arrested by simple pressure treatment, electrocautery, and H_2_O_2_ painting. To control postoperative pain, patients will be equipped with a patient-controlled anesthesia device (PCA; Aim Plus, Abbott Laboratories, Illinois) to deliver fentanyl. If a patient wants additional analgesics after the operation, intramuscular injections of ketorolac tromethamine 30 mg will be provided. In addition, as a basic analgesic, acetaminophen will be administered for 7 days after the operation. In other words, you will receive the same operation and postoperative care as you would if you chose to participate in the study.

## 11. Personal information protection

Your personal information, including study participation records and medical records, will be managed so that it is only provided to the person in charge of the review of such information. However, it is difficult to guarantee that your data will be kept completely confidential. Related agencies, such as the KFDA or the Ministry of Health and Welfare, may browse your data and make copies of them. The study results may be published in the future, but your name and other personal information will be kept strictly confidential.

## 12. Discontinuation of participation in the clinical study

You may discontinue your participation in the study whenever you want without any disadvantage or loss of benefit. If you want to stop participating in the study at any time, please contact the investigator. The investigator will then conduct the discontinuation process.

The investigator may drop you from the clinical study at any time without your consent if he or she judges that discontinuation is the best choice for you, or if you violate the participant compliance criteria (e.g. if you do not complete the questionnaire). In addition, the sponsor of this clinical study may discontinue the entire study.

If new information that affects your health and welfare or continuing participation in the study becomes available, we will inform you.

If you acquire any injury or disease as a result of participating in this study, you will receive appropriate medical treatment. Depending on the situation, this treatment may be free of charge. If you need more information, please contact the investigator.

**Consent Form**

| ***The effectiveness of pregabalin for post-tonsillectomy pain control***  Your signature below indicates that you consent to participate in this study, and your protected health information will be exposed and used. | | |
| --- | --- | --- |
|  |  | |
| Name of subject |  |  |
|  |  |  |
| Signature of subject |  | Date |
| **Do not sign after this date.** | 🡪 |  |
|  |  |  |
| Signature of person who obtains consent |  | Date |
|  |  | |
| Name of person who obtains consent |  |  |
| Date of preparation: |  | |

**18. Regulations on subject compensation**

In principle, there is no monetary reward for participation in this clinical study.

**19. Subjects’ treatment, treatment criteria, and safety protection measures after the clinical study**

The subjects will receive progress observation and treatment in the outpatient clinic for 2 weeks after tonsillectomy. Also, after discharge, basic daily analgesics will be allowed for both groups of patients equally. According to previous studies, except for drowsiness, no side effects, after effects, or complications because of the administration pregabalin before the operation are expected to occur. Nevertheless, the principal investigator will ensure the safety and protection of subjects during the clinical study, and if serious adverse events occur, they will be reported to the IRB.

**20. Actions for safety and protection of subjects**

The principal investigator and coinvestigator will ensure the safety and protection of all patient participants, and if serious adverse events occur, they will be reported to the IRB.

**21. Case report form**

●Postoperative Pain Record (for patient)

Dear Patient,

We assume that you are concerned about your tonsillectomy.

Tonsillectomy is a type of surgery where the surgical site is exposed, and pain occurs when talking or eating. To manage the pain in patients after tonsillectomy a little better, the Department of Otolaryngology in our hospital conducts a survey of your pain after tonsillectomy. Because this survey is conducted for more effective pain control, please respond truthfully. We appreciate your cooperation. **If your pain becomes worse during your stay in hospital, please ask the attending physicians or nurses to give you additional analgesic (painkiller) whenever you need it.**

1. For the degree of pain:

0 means you do not feel pain at all, 1–2 points means you are uncomfortable, 3–4 points means you feel a little pain, 5–6 points means you feel a lot of pain, 7–8 points means it is very painful, and 9–10 points means the most intense pain that can be imagined (as if your arms and legs had been chopped off).

# 1 hour after surgery ( : )

| Degree of Pain | 0 | 1 | 2 | 3 | 4 | 5 | 6 | 7 | 8 | 9 | 10 |
| --- | --- | --- | --- | --- | --- | --- | --- | --- | --- | --- | --- |
| When staying still |  | | | | | | | | | | |
| When swallowing saliva |  | | | | | | | | | | |

# 2 hours after surgery ( : )

| Degree of Pain | 0 | 1 | 2 | 3 | 4 | 5 | 6 | 7 | 8 | 9 | 10 |
| --- | --- | --- | --- | --- | --- | --- | --- | --- | --- | --- | --- |
| When staying still |  | | | | | | | | | | |
| When swallowing saliva |  | | | | | | | | | | |

# 4 hour after surgery ( : )

| Degree of Pain | 0 | 1 | 2 | 3 | 4 | 5 | 6 | 7 | 8 | 9 | 10 |
| --- | --- | --- | --- | --- | --- | --- | --- | --- | --- | --- | --- |
| When staying still |  | | | | | | | | | | |
| When swallowing saliva |  | | | | | | | | | | |

# 8 hours after surgery ( : )

| Degree of Pain | 0 | 1 | 2 | 3 | 4 | 5 | 6 | 7 | 8 | 9 | 10 |
| --- | --- | --- | --- | --- | --- | --- | --- | --- | --- | --- | --- |
| When staying still |  | | | | | | | | | | |
| When swallowing saliva |  | | | | | | | | | | |

# 12 hour after surgery ( : )

| Degree of Pain | 0 | 1 | 2 | 3 | 4 | 5 | 6 | 7 | 8 | 9 | 10 |
| --- | --- | --- | --- | --- | --- | --- | --- | --- | --- | --- | --- |
| When staying still |  | | | | | | | | | | |
| When swallowing saliva |  | | | | | | | | | | |

# 24 hours after surgery ( : )

| Degree of Pain | 0 | 1 | 2 | 3 | 4 | 5 | 6 | 7 | 8 | 9 | 10 |
| --- | --- | --- | --- | --- | --- | --- | --- | --- | --- | --- | --- |
| When staying still |  | | | | | | | | | | |
| When swallowing saliva |  | | | | | | | | | | |

**2. From after surgery to discharge, how satisfied are you with overall pain control?**

(0 points means you are not satisfied at all, and 10 points means you are satisfied completely.)

| Degree of Satisfaction | 0 | 1 | 2 | 3 | 4 | 5 | 6 | 7 | 8 | 9 | 10 |
| --- | --- | --- | --- | --- | --- | --- | --- | --- | --- | --- | --- |
| When staying still |  | | | | | | | | | | |

**3. Please tell us your other symptoms.**

# I felt sick in the stomach and felt like vomiting.---- Not at all ( )

A little bit ( )

Very much ( )

# I vomited.--------------------- Not at all ( )

A little bit ( )

Very much ( )

# I felt dizzy.-------------------- Not at all ( )

A little bit ( )

Very much ( )

# I felt like sleeping, although I did not want or expect to do so.----

Not at all ( )

A little bit ( )

Very much ( )

# I had a headache.------------------ Not at all ( )

A little bit ( )

Very much ( )

**●Pain Record after Discharge (for patient)**

1. For the degree of pain:

0 means you do not feel pain at all, 1–2 points means you are uncomfortable, 3–4 points means you feel a little pain, 5–6 points means you feel a lot of pain, 7–8 points means it is very painful, and 9–10 points means the most intense pain that can be imagined (as if your arms and legs had been chopped off).

# Before breakfast on day 1 after discharge ( day)

| Degree of Pain | 0 | 1 | 2 | 3 | 4 | 5 | 6 | 7 | 8 | 9 | 10 |
| --- | --- | --- | --- | --- | --- | --- | --- | --- | --- | --- | --- |
| When staying still |  | | | | | | | | | | |
| When swallowing saliva |  | | | | | | | | | | |

# Before breakfast on day 2 after discharge ( day)

| Degree of Pain | 0 | 1 | 2 | 3 | 4 | 5 | 6 | 7 | 8 | 9 | 10 |
| --- | --- | --- | --- | --- | --- | --- | --- | --- | --- | --- | --- |
| When staying still |  | | | | | | | | | | |
| When swallowing saliva |  | | | | | | | | | | |

# Before breakfast on day 3 after discharge ( day)

| Degree of Pain | 0 | 1 | 2 | 3 | 4 | 5 | 6 | 7 | 8 | 9 | 10 |
| --- | --- | --- | --- | --- | --- | --- | --- | --- | --- | --- | --- |
| When staying still |  | | | | | | | | | | |
| When swallowing saliva |  | | | | | | | | | | |

# Before breakfast on day 4 after discharge ( day)

| Degree of Pain | 0 | 1 | 2 | 3 | 4 | 5 | 6 | 7 | 8 | 9 | 10 |
| --- | --- | --- | --- | --- | --- | --- | --- | --- | --- | --- | --- |
| When staying still |  | | | | | | | | | | |
| When swallowing saliva |  | | | | | | | | | | |

# Before breakfast on day 5 after discharge ( day)

| Degree of Pain | 0 | 1 | 2 | 3 | 4 | 5 | 6 | 7 | 8 | 9 | 10 |
| --- | --- | --- | --- | --- | --- | --- | --- | --- | --- | --- | --- |
| When staying still |  | | | | | | | | | | |
| When swallowing saliva |  | | | | | | | | | | |

# Before breakfast on day 6 after discharge ( day)

| Degree of Pain | 0 | 1 | 2 | 3 | 4 | 5 | 6 | 7 | 8 | 9 | 10 |
| --- | --- | --- | --- | --- | --- | --- | --- | --- | --- | --- | --- |
| When staying still |  | | | | | | | | | | |
| When swallowing saliva |  | | | | | | | | | | |

# Before breakfast on day 7 after discharge ( day)

| Degree of Pain | 0 | 1 | 2 | 3 | 4 | 5 | 6 | 7 | 8 | 9 | 10 |
| --- | --- | --- | --- | --- | --- | --- | --- | --- | --- | --- | --- |
| When staying still |  | | | | | | | | | | |
| When swallowing saliva |  | | | | | | | | | | |

**2. From after surgery to now, how satisfied are you with overall pain control?**

(0 points means you are not satisfied at all, and 10 points means you are satisfied completely.)

| Degree of Satisfaction | 0 | 1 | 2 | 3 | 4 | 5 | 6 | 7 | 8 | 9 | 10 |
| --- | --- | --- | --- | --- | --- | --- | --- | --- | --- | --- | --- |
| When staying still |  | | | | | | | | | | |

**●Adult Tonsillectomy Information Record (for investigator)**

No:

Sex: Age:

Height: Weight:

Operation Date: Name of Operation:

Operation Time: Anesthesia Time:

# Use of additional analgesic and number of times:

# Amount of use of PCA ( ) ml

**22. Other necessities for safe and scientific conduct of the clinical study**

Not applicable

**23. Clinical literature that can be used as a source for the current study (references)**

1)[Agarwal A](http://www.ncbi.nlm.nih.gov/pubmed?term=%22Agarwal%20A%22%5BAuthor%5D&itool=EntrezSystem2.PEntrez.Pubmed.Pubmed_ResultsPanel.Pubmed_RVAbstract), [Gautam S](http://www.ncbi.nlm.nih.gov/pubmed?term=%22Gautam%20S%22%5BAuthor%5D&itool=EntrezSystem2.PEntrez.Pubmed.Pubmed_ResultsPanel.Pubmed_RVAbstract), [Gupta D](http://www.ncbi.nlm.nih.gov/pubmed?term=%22Gupta%20D%22%5BAuthor%5D&itool=EntrezSystem2.PEntrez.Pubmed.Pubmed_ResultsPanel.Pubmed_RVAbstract), [Agarwal S](http://www.ncbi.nlm.nih.gov/pubmed?term=%22Agarwal%20S%22%5BAuthor%5D&itool=EntrezSystem2.PEntrez.Pubmed.Pubmed_ResultsPanel.Pubmed_RVAbstract), [Singh PK](http://www.ncbi.nlm.nih.gov/pubmed?term=%22Singh%20PK%22%5BAuthor%5D&itool=EntrezSystem2.PEntrez.Pubmed.Pubmed_ResultsPanel.Pubmed_RVAbstract), [Singh U](http://www.ncbi.nlm.nih.gov/pubmed?term=%22Singh%20U%22%5BAuthor%5D&itool=EntrezSystem2.PEntrez.Pubmed.Pubmed_ResultsPanel.Pubmed_RVAbstract). Evaluation of a single preoperative dose of pregabalin for attenuation of postoperative pain after laparoscopic cholecystectomy. [Br J Anaesth.](javascript:AL_get(this,%20'jour',%20'Br%20J%20Anaesth.');) 2008 Nov; 101(5):700-4.

2) Mathiesen O, Jacobsen LS, Holm HE, Randall S, Adamiec-Malmstroem L, Graungaard BK, Holst PE, Hilsted KL, Dahl JB. [Pregabalin and dexamethasone for postoperative pain control: a randomized controlled study in hip arthroplasty.](http://www.ncbi.nlm.nih.gov/pubmed/18653493?itool=EntrezSystem2.PEntrez.Pubmed.Pubmed_ResultsPanel.Pubmed_RVDocSum&ordinalpos=2) Br J Anaesth. 2008 Oct;101(4):535-41.

3) Mathiesen O, Rasmussen ML, Dierking G, Lech K, Hilsted KL, Fomsgaard JS, Lose G, Dahl JB. Pregabalin and dexamethasone in combination with paracetamol for postoperative pain control after abdominal hysterectomy. A randomized clinical trial. Acta Anaesthesiol Scand. 2009 Feb;53(2):227-35.

4) Freedman BM, O'Hara E. [Pregabalin has opioid-sparing effects following augmentation mammaplasty.](http://www.ncbi.nlm.nih.gov/pubmed/19083556?itool=EntrezSystem2.PEntrez.Pubmed.Pubmed_ResultsPanel.Pubmed_RVDocSum&ordinalpos=1) Aesthet Surg J. 2008 Jul-Aug;28(4):421-4.

5) Jokela R, Ahonen J, Tallgren M, Haanpää M, Korttila K. [Premedication with pregabalin 75 or 150 mg with ibuprofen to control pain after day-case gynaecological laparoscopic surgery.](http://www.ncbi.nlm.nih.gov/pubmed/18448418?itool=EntrezSystem2.PEntrez.Pubmed.Pubmed_ResultsPanel.Pubmed_RVDocSum&ordinalpos=2) Br J Anaesth. 2008 Jun;100(6):834-40.

6) Ha SL, Kang BH, Lee SH, Kim SY. Postoperative results in adult patients after tonsillectomy. Korean J Otolaryngol-Head Neck Surg 1999;42:1279-83.

7) Moiniche S, Romsing J, Dahl JB, Tramer MR. Nonsteroidal antiinflammatory drugs and the risk of operative site bleeding after tonsillectomy: a quantitative systematic review. Anesth Analg 2003;96:68–77.

8) Marret E, Flahault A, Samama CM, Bonnet F. Effects of postoperative nonsteroidal antiinflammatory drugs on bleeding risk after tonsillectomy.

Anesthesiology 2003;98:1497–502.

9) Grass JA, Sakima NT, Valley M, Fisher K, Jackson C, Walsh P, et al. Assessment of ketorolac as an adjuvant to fentanyl patient-controlled epidural analgesia after radical retropubic prostatectomy. Anesthesiology 1993;78:642-8.

10) Cho KS, Choi CJ, Im SK, Cha HE. Scuralfate and Guaiazulene in alleviating post-tonsillectomy morbidity. Korean J Otolaryngol-Head Neck Surg 1994;37:998-1003.

11) Carr DB, Goudas LC. Acute pain. Lancet 1999; 353: 2051–58.

12) Coderre TJ, Katz J, Vaccarino AL, Melzack R. Contribution of central neuroplasticity to pathological pain: review of clinical and experimental evidence. Pain 1993;52:259-85.

13) Goa KL, Sorkin EM. Gabapentin: A review of its pharmacological properties and clinical potential in epilepsy. Drug 1993;46:409-47.

14) Woolf CJ, Chong MS. Preemptive analgesia-treating postoperative pain by preventing the establishment of central sensitization. Anesth Analg 1993; 77: 362–79.
